# Early gastric cancer with malignant invasion into the duodenum: A case report

**DOI:** 10.1097/MD.0000000000047082

**Published:** 2026-01-16

**Authors:** Hao Fu, AiFeng Pan, XiaoYan Han, JianJun Qu, GuangXu Zhu, XuGuang Jiao, XiaoMin Lang

**Affiliations:** a Department of Gastrointestinal Surgery Medical Center, Weifang People’s Hospital, Weifang, Shandong, China.

**Keywords:** duodenum invasion, early gastric cancer, narrow-band imaging magnification endoscopy, pathophysiology, surgical management

## Abstract

**Rationale:**

Well-differentiated early gastric cancer (EGC) typically exhibits limited invasive potential, and duodenal involvement is exceedingly rare. This report presents a unique case of a well-differentiated early gastric cancer with extensive duodenal invasion. Magnifying endoscopy is critical for elucidating the growth and infiltration patterns of such lesions. Furthermore, this study provides a detailed magnified endoscopic comparison between the gastric and duodenal mucosa.

**Patient concerns:**

A 62-year-old female patient presented with 20 days of epigastric discomfort, but no nausea, vomiting or difficulty eating. Gastroscopy was performed to clarify the diagnosis, revealing circumferential thickening of the distal gastric sinus that continued across the pylorus to the duodenum. The patient was subsequently hospitalized for further endoscopic examination and pathological biopsy, and was diagnosed with early differentiated gastric cancer that had invaded the duodenum.

**Diagnoses:**

Early gastric cancer with duodenum invasion.

**Interventions:**

laparoscopic-assisted major gastrectomy with Billroth II anastomosis and D2 lymph node dissection.

**Outcomes:**

The patient underwent successful surgery and was discharged after 9 days of postoperative rehabilitation.

**Lessons:**

This case provides evidence to support the idea that early gastric cancer can spread to the duodenum by directly penetrating the pylorus. It also presents magnified endoscopic images and histopathological analyses that have not been shown in previous reports.

## 1. Introduction

Early gastric cancer (EGC) is defined as malignant lesions confined to the gastric mucosal layer and submucosa, regardless of lesion size or lymph node metastasis status. Well-differentiated adenocarcinomas exhibit lower invasive potential and demonstrate indolent progression. It has been reported that 11.9% to 23.8% of patients with gastric antral cancer are diagnosed with tumor infiltration into the duodenum, with the majority of such cases occurring during advanced stages of disease progression.^[1]^ Transpyloric invasion of EGC into the duodenum remains exceptionally rare.

Herein, we present a case of differentiated EGC in the gastric antrum demonstrating extensive duodenal involvement. This case distinguishes itself from previously reported instances through 3 principal features: the large-scale duodenal invasion (>2 cm), atypical gross findings (laterally spreading), and comprehensive documentation through detailed, magnified endoscopic observations. Therefore, we report this unusual presentation of EGC along with a literature review.

## 2. Narrative

### 2.1. Patient information

A 62-year-old Han female presented to the outpatient department with a 20-day history of upper abdominal discomfort, without symptoms such as nausea, vomiting, reflux, or difficulty in eating. The abdominal and physical examinations did not reveal any signs of abdominal pain, nor other irregularities, although she had over a 3-year history of hypertension that had been effectively managed through a standardized medication. A subsequent gastroscopy examination revealed an area of circumferential thickening around the gastric antrum and an encroachment of the duodenal bulb across the pyloric ring. The biopsy of the intragastric lesion further confirmed a high-grade intraepithelial neoplasia (HGIN). Laboratory tests including blood routine, biochemical routine, and gastrointestinal tumor markers did not reveal any abnormalities. Serological testing confirmed Helicobacter pylori positivity, despite prior eradication therapy administered 2 years earlier.

### 2.2. Timeline

**Figure 1.**
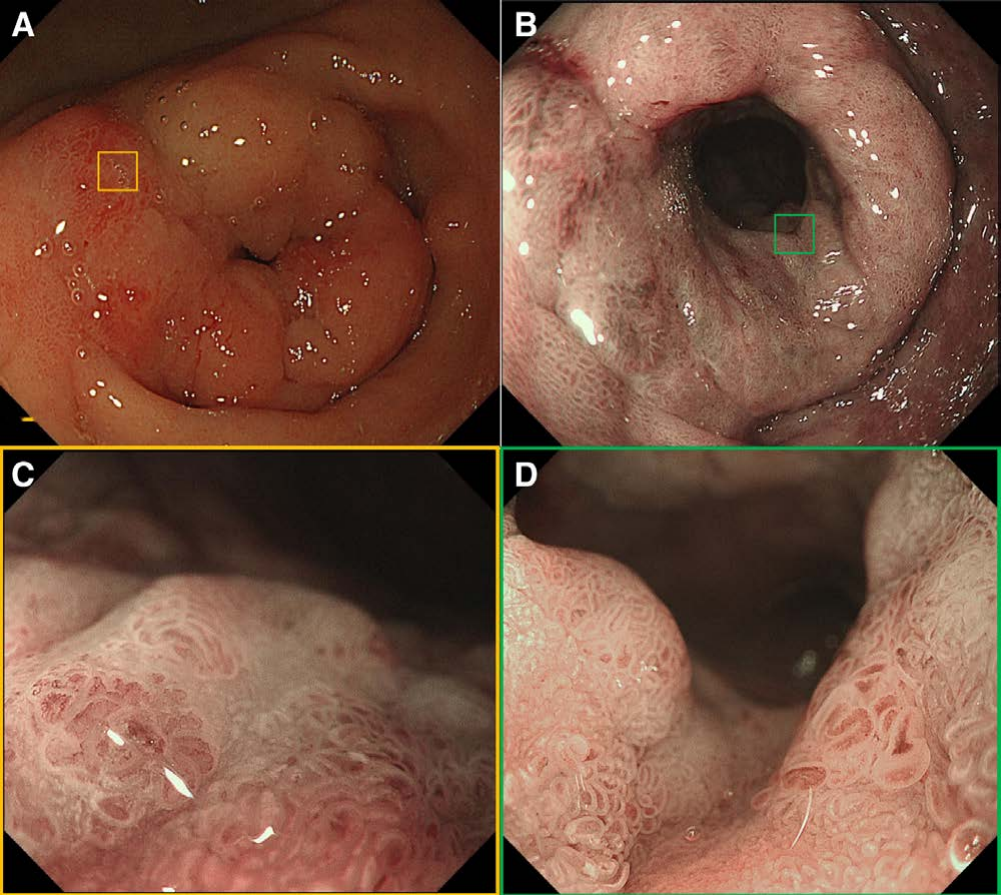
The distal gastric antrum and peripyloric ring display a heterogeneous thickening characterized by an irregularly elevated surface (0-I + 0-IIa) (A). The lesion exhibits the softness during the endoscopic insufflation test (B). NBI-ME demonstrates that the overall mucosa of the lesion displays a distinct brownish-colored and irregularly-widened conduit, and the lesion surface is covered with WOS (C and D).

**Figure 2.**
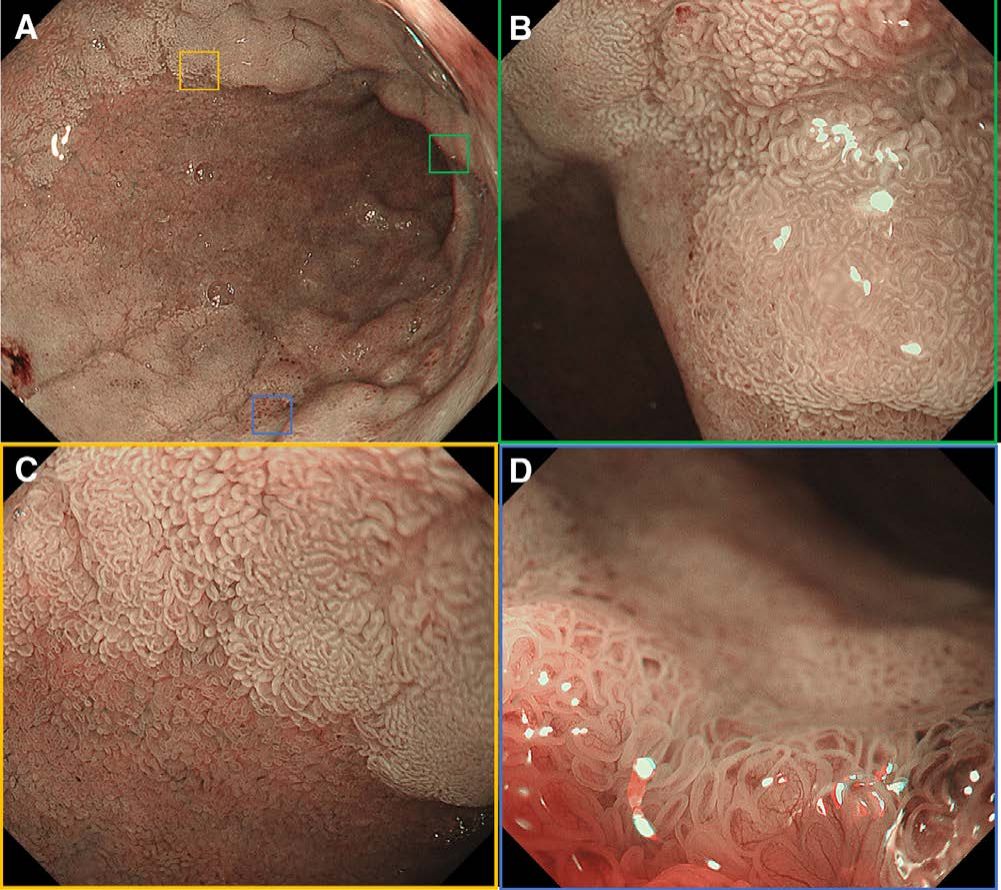
The duodenal bulb lesion exhibits a flatly elevated shape that is a direct extension of the gastric sinus lesion characterized by clear demarcations and extensive smears of WOS (A–C). Upon a close examination, the lesion displays IMVP and IMSP (D).

*2.2.1. Diagnostic assessment*: Intensive magnification endoscopy revealed thickening of the distal gastric antrum and pyloric ring with an uneven elevated lesion (Paris classification 0–IIa) (Fig. 1A and B). Narrow-band imaging magnification endoscopy (NBI-ME) identified atrophic, distorted, and brownish mucosa in the gastric antrum. Irregularly widened and disoriented glandular ducts were observed, accompanied by distorted periductal vasculature and diffuse coverage by white opaque substance (Fig. 1C and D). Endoscopic insufflation demonstrated a well-defined lesion extending into the duodenal bulb, exhibiting a flat-elevated morphology (0–IIa) with sharp margins, resembling a laterally spreading tumor (Fig. 2A). NBI-ME further revealed extensive white opaque substance coverage, irregular microvascular patterns (IMVP), and microsurface patterns (IMSP), with vessels displaying heterogeneous caliber and disorganized alignment (Fig. 2B–D). Notably, duodenal mucosal distortion was less severe compared to the gastric antrum. Enhanced abdominal computed tomography showed marginal gastric antral thickening without lymphadenopathy or distant metastasis. Biopsies confirmed HGIN in the gastric antrum and focal duodenal lesions, with low-grade intraepithelial neoplasia in the duodenum.

**Figure 3.**
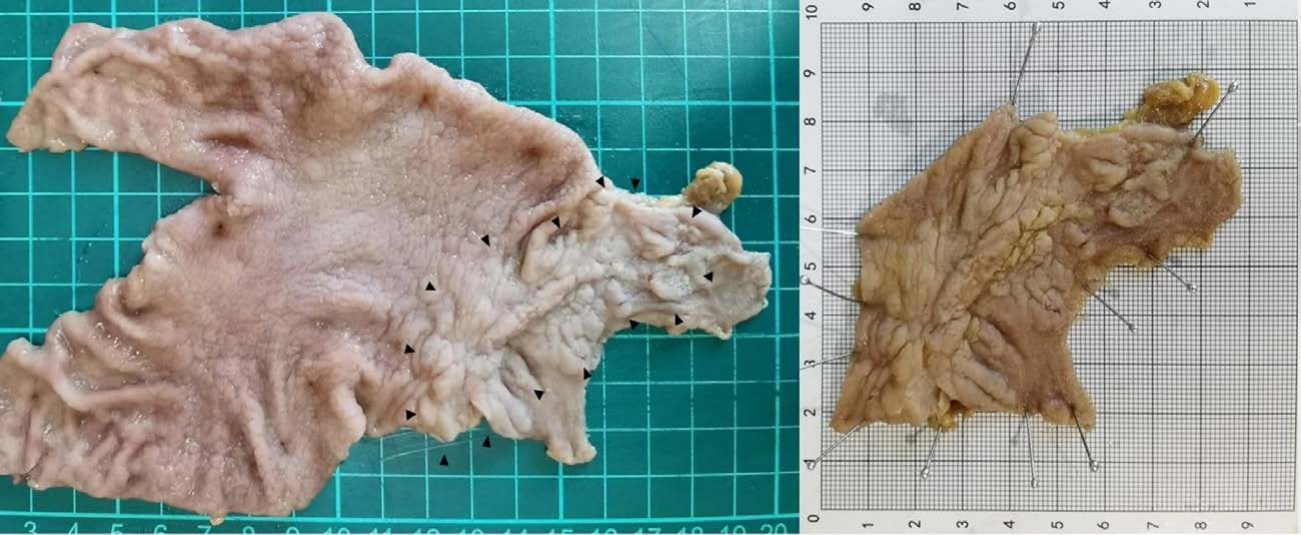
A macroscopic examination of the resected specimen showing an irregular flatly elevated lesion spreading from the gastric antrum to the duodenum beyond the pyloric ring.

**Figure 4.**
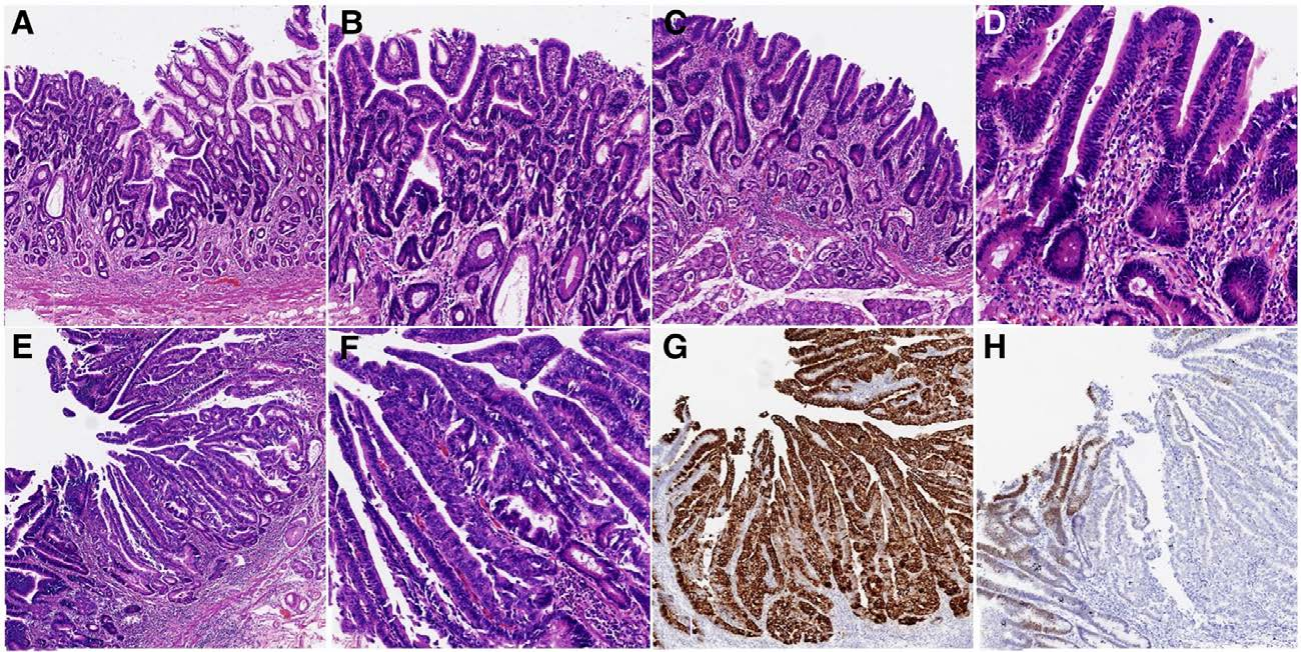
The surgical specimen reveal the presence of HGIN in the gastric antrum region characterized by a disrupted glandular duct-shaped structure and the loss of polarity without penetrating into the muscular layer of the mucosa at the 100X magnification (A). At a high magnification (200X), the observed nuclei are rod-shaped and display intensely-stained and stacked multiple layers, but these cells maintain their inherent cell polarity (B). The irregularly shaped duodenal glands characterized by villous changes are noticeable with an intact muscular layer of the mucosa with a general appearance of HGIN (C). With the 300x magnification, the crowded and rod-shaped nuclei, abnormal cytoplasmic ratio, and massive inflammatory cell infiltration in the interstitial connective tissue can be seen (D). The moderately differentiated adenocarcinoma is located within the thickened tissue of the pyloric ring, suggesting a poor differentiation (E and F). Immunostains suggests that the lesion as a whole is strongly positive for Muc5ac and partially positive for CDX2 (G and H). HGIN = high-grade intraepithelial neoplasia.

*2.2.2. Therapeutic intervention:* A multidisciplinary team consensus classified the lesion as EGC. Given the lesion’s circumferential involvement of the pylorus and duodenum (>6 cm), endoscopic submucosal dissection was deemed high-risk due to anticipated complications (stenosis, perforation). laparoscopic-assisted major gastrectomy with Billroth II anastomosis and D2 lymph node dissection was performed. Postoperative histopathology confirmed well-differentiated adenocarcinoma in the gastric antrum (confined to the muscularis mucosa layer) and low-grade intraepithelial neoplasia in the duodenum (Fig. 3). Tumor dimensions measured 6.5 × 4.6 cm, with duodenal extension and a 2.6 cm anal margin from the pylorus. Resection margins were tumor-free, and no lymphatic/venous invasion or nodal metastasis (pT1aN0M0, stage Ia) was observed. Histopathological analysis of the surgical specimen confirmed HGIN coexisting with localized moderately differentiated adenocarcinoma (Fig. 4A–F). Immunohistochemistry (IHC) demonstrated diffuse strong positivity for MUC5AC (+++) and focal CDX2 expression (+) within the lesion (Fig. 4G and H). P53 exhibited weak nuclear staining (1+) in 30% of tumor cells, while the Ki-67 proliferative index reached approximately 60%.

*2.2.3. Limitations:* The patient did not undergo EUS due to its unavailability at the time, precluding more detailed assessment. Additionally, although IHC for MUC5AC and CDX2 was performed, expanding the panel to include MUC6 and CD20 would have been beneficial for a more complete pathological interpretation.

*2.2.4. Follow-up and outcomes:* Postoperative recovery was uneventful. She was well after postoperative 2-year follow-up. The timeline table is presented in Table 1.

**Table 1:** Case summary and treatment timeline.

Date	Event	Key Findings
May 4, 2023	Initial visit to the Gastroenterology outpatient clinic due to “epigastric dull pain for 20 days”	Physical examination revealed no significant abnormalities. Gastroscopy was recommended.
May 6, 2023	Gastroscopy examination	A mucosal lesion in the pyloric region extending into the duodenum was identified.
May 7, 2023	Admitted to the hospital for further diagnostic workup	Blood routine test and tumor markers showed no abnormalities. Enhanced abdominal CT revealed a nonsignificantly enhancing lesion in the antral region with no peri-gastric lymphadenopathy (cT1N0M0).
May 11, 2023	Magnification endoscopy examination	The lesion was pliable. Irregular Microsurface Pattern and Irregular Microvascular Pattern were observed, consistent with stage cT1a to cT1b.
May 18, 2023	Underwent totally laparoscopic radical gastrectomy with D2 lymph node dissection (B2 reconstruction)	The procedure was successful, and the tumor was completely resected.
May 23, 2023	Pathological report	Findings indicated gastric high-grade intraepithelial neoplasia, low-grade intraepithelial neoplasia, with a focal area of well-differentiated adenocarcinoma (pT1aN0M0, Stage I).
May 31, 2023	Discharged from the hospital	The patient’s general condition was good, with well-healed wounds and tolerating a semiliquid diet.
June 2023–Present	Follow-up	No recurrence has been detected.

CT = computed tomography.

## 3. Patient perspective

The patient initially expressed a strong preference for endoscopic resection as a less invasive treatment option. However, after detailed consultation with both the surgical and gastroenterology teams, they understood that due to the lesion’s specific location and characteristics, laparoscopic surgery was necessary to achieve complete oncological resection. Postoperatively, the patient reported a satisfactory recovery course. They have experienced no major complications, such as reflux gastritis or esophagitis, and are pleased that their overall quality of life has been maintained without significant deterioration.

## 4. Discussion

Gastric cancer pathogenesis involves complex genetic and environmental interactions. Patients with EGC often remain asymptomatic, as demonstrated in this case where a distal gastric tumor involving the pyloric ring presented solely with abdominal bloating rather than typical malignancy symptoms. This underscores endoscopy’s critical role in early detection. While advanced sinus cancers frequently exhibit submucosal duodenal invasion, EGC with duodenal involvement remains exceptionally rare. Namikawa et al^[2]^ identified only 13 such cases over 35 years, with average tumor diameter 6.3 cm and duodenal infiltration length 0.9 cm. Most cases involved differentiated (Tub) or signet ring cell (Sig) cancers. Our case exhibited Tub1 histology with gastric-type differentiation via immunohistochemistry, demonstrating duodenal invasion exceeding previously reported dimensions. A Medline review revealed only 4 relevant reports in 2 decades,^[2–4]^ whereas our documentation provides comprehensive magnified endoscopic images detailing both macroscopic morphology and microvascular patterns in flat/depressed areas. Endoscopically, the lesion displayed characteristic features of differentiated EGC. The gastric component appeared as a flat-elevated type 0-IIa lesion with a well-demarcated margin, IMSP and IMVP. Importantly, no high-risk endoscopic indicators of deep submucosal invasion – such as ulceration or markedly dilated vessels – were observed. The tumor extended across the pylorus into the duodenum while preserving the 0-IIa morphological pattern. Although the duodenal segment exhibited more regular pit architecture compared to the gastric portion, it still showed mild cytological and architectural atypia relative to the adjacent normal duodenal mucosa. Immunohistochemical analysis revealed strong MUC5AC expression, supporting a gastric phenotypic origin. Histopathologically, the lesion was predominantly consistent with low-grade to HGIN, with only a focal area in the gastric region demonstrating well-differentiated adenocarcinoma. The extensive circumferential involvement of both the distal stomach and proximal duodenum is exceedingly rare in the context of well-differentiated EGC, and the biological mechanisms driving this unusual growth pattern remain poorly understood.

The gastroduodenal junction lacks clear anatomical demarcation, with the pyloric ring serving as a clinical reference point.^[5]^ Pathologically, Brunner gland emergence typically defines the duodenal boundary.^[6]^ The lower incidence of malignancies at this junction compared to the esophagogastric junction raises questions about pyloric microstructure’s protective role.Notably, a Japanese study^[7]^ reported EGC recurrence with duodenal invasion 8 months post-EMR, suggesting acquired pyloric mucosal disruption may facilitate tumor spread. This observation prompts investigation into potential mucosal-submucosal barriers inhibiting distal tumor progression. Histopathologic analyses consistently demonstrate preserved Brunner gland architecture despite duodenal mucosal infiltration by gastric adenocarcinoma.^[8]^ This structural resilience supports the hypothesis that Brunner gland organization may constitute a morphological barrier against transpyloric tumor progression. Mechanistically, their secretory products – including epidermal growth factor and amphipathic lipids – may modulate mucosal repair and inhibit cancer cell adhesion.^[9]^ However, the lack of consensus on their tumor-suppressive role underscores the need for functional studies correlating gland density with invasion risk.

From a molecular pathology standpoint, it has been proposed that the demarcation between gastric and duodenal regions lies in the expression boundary of SOX2 and CDX2.^[10]^ Meanwhile, abnormal CDX2 expression in the stomach is believed to be associated with intestinal metaplasia, which in turn is closely related to the development of intestinal-type gastric cancer, providing evidence for the molecular expression boundary theory. The SOX2/CDX2 expression boundary theory requires further integration with genomic instability for comprehensive interpretation. A landmark 2025 Lancet review highlighted that diffuse-type gastric cancer (frequently accompanied by CDH1/RHOA mutations) is more prone to transregional infiltration, which may be associated with CDX2 expression dysregulation mediated by epigenetic mechanisms.^[11]^ The Asian Cancer Research Group’s TP53 activity-based classification further suggests that aberrant CDX2 expression in TP53-inactive tumors correlates positively with intestinal metaplasia progression, supporting the notion that molecular boundary failure may be a critical step in the infiltration of intestinal-type gastric cancer into the duodenum.^[12]^

## 5. Conclusion

This case is an excellent addition to EGC involving the stomach and duodenum. The previously reported case did not have an adequate magnified endoscopic view. Such cross-regional extension of EGC is rare, and further investigation of additional cases is needed to unravel the underlying mechanism of EGC with duodenal invasion. This case has revealed that the EGC lesion adjacent to the gastric antrum, particularly to the prepyloric region, possess the ability to directly penetrate the duodenal mucosal layer. Concurrently, care should be taken during endoscopic procedures near the pyloric region to avoid iatrogenic damage to the mucosal barrier.

## Author contributions

**Data curation:** AiFeng Pan, GuangXu Zhu.

**Investigation:** XiaoYan Han.

**Methodology:** XuGuang Jiao, XiaoMin Lang.

**Project administration:** XiaoMin Lang.

**Resources:** GuangXu Zhu.

**Validation:** JianJun Qu.

**Writing – original draft:** Hao Fu.

**Writing – review & editing:** JianJun Qu.
